# The efficacy and neural mechanism of acupuncture therapy in the treatment of visceral hypersensitivity in irritable bowel syndrome

**DOI:** 10.3389/fnins.2023.1251470

**Published:** 2023-09-04

**Authors:** Yuanzhen Yang, Jiaqi Wang, Chaoyang Zhang, Yi Guo, Meidan Zhao, Man Zhang, Zhongzheng Li, Feifei Gao, Yu Luo, Yiru Wang, Junyi Cao, Mingfang Du, Yuzhe Wang, Xiaowei Lin, Zhifang Xu

**Affiliations:** ^1^Research Center of Experimental Acupuncture Science, Tianjin University of Traditional Chinese Medicine, Tianjin, China; ^2^National Clinical Research Center for Chinese Medicine Acupuncture and Moxibustion, Tianjin, China; ^3^School of Traditional Chinese Medicine, Tianjin University of Traditional Chinese Medicine, Tianjin, China; ^4^Tianjin Key Laboratory of Modern Chinese Medicine Theory of Innovation and Application, Tianjin, China; ^5^School of Medical Technology, Tianjin University of Traditional Chinese Medicine, Tianjin, China; ^6^School of Acupuncture & Moxibustion and Tuina, Tianjin University of Traditional Chinese Medicine, Tianjin, China

**Keywords:** acupuncture, analgesia, irritable bowel syndrome, visceral hypersensitivity, neural mechanism

## Abstract

Irritable Bowel Syndrome (IBS) is a complex functional gastrointestinal disorder primarily characterized by chronic abdominal pain, bloating, and altered bowel habits. Chronic abdominal pain caused by visceral Hypersensitivity (VH) is the main reason why patients with IBS seek medication. Significant research effort has been devoted to the efficacy of acupuncture as a non-drug alternative therapy for visceral-hyperalgesia-induced IBS. Herein, we examined the central and peripheral analgesic mechanisms of acupuncture in IBS treatment. Acupuncture can improve inflammation and relieve pain by reducing 5-hydroxytryptamine and 5-HT3A receptor expression and increasing 5-HT4 receptor expression in peripheral intestinal sensory endings. Moreover, acupuncture can also activate the transient receptor potential vanillin 1 channel, block the activity of intestinal glial cells, and reduce the secretion of local pain-related neurotransmitters, thereby weakening peripheral sensitization. Moreover, by inhibiting the activation of *N*-methyl-D-aspartate receptor ion channels in the dorsal horn of the spinal cord and anterior cingulate cortex or releasing opioids, acupuncture can block excessive stimulation of abnormal pain signals in the brain and spinal cord. It can also stimulate glial cells (through the P2X7 and prokinetic protein pathways) to block VH pain perception and cognition. Furthermore, acupuncture can regulate the emotional components of IBS by targeting hypothalamic-pituitary-adrenal axis-related hormones and neurotransmitters via relevant brain nuclei, hence improving the IBS-induced VH response. These findings provide a scientific basis for acupuncture as an effective clinical adjuvant therapy for IBS pain.

## 1. Introduction

Irritable bowel syndrome (IBS) is a functional intestinal disorder characterized by abdominal pain or discomfort associated with changes in bowel habits and stool characteristics. The yearly increase in IBS incidence can be attributed to modern development and lifestyle changes. According to the global meta-analysis, IBS affects an estimated 5–22% of the general population. The latest Rome IV criteria (Mearin et al., 2016), classifies IBS patients into four subtypes, i.e., Diarrheal IBS (IBS-D), Constipated IBS (IBS-C), Mixed IBS (IBS-M) and Unclassifiable IBS (IBS-U), among which IBS-D is the most prevalent at 40% (Lovell and Ford, 2012b). Considering their severe impact on patient quality of life, the complex and diverse clinical IBS manifestations are increasingly attracting research attention. Visceral pain, not frequent diarrhea, is the primary IBS symptom and the major reason why patients seek treatment (Yu et al., 2021). Unlike other symptoms, visceral pain as an IBS disease marker is the most severe symptom of the disease (Spiegel et al., 2010). One of the effective approaches to manage IBS pain hypersensitivity is to increase the pain threshold or reduce pain sensitivity (Nee and Lembo, 2021). Nonetheless, no universally accepted treatment prevents pain progression, since the visceral analgesic drug effects including 5-HT3 receptor antagonists (alosetron, ondansetron, ramosetron), non-absorbable antibiotics (rifaximin), and secretagogues (lubiprostone, linaclotide) are insufficient and cannot meet clinical treatment needs (Camilleri and Boeckxstaens, 2017).

In a clinical context, acupuncture is a general term comprising many therapeutic procedures, including traditional human acupuncture, moxibustion, electric acupuncture, and laser acupuncture, among other techniques used in the analgesic treatment of clinically relevant acute and chronic pain diseases, such as an acute sprain, migraine, osteoarthritis, labor pain, cancer pain, etc. (Dang and Yang, 1998; Cohen et al., 2011; Mackenzie et al., 2011). A recent meta-analysis of randomized controlled clinical trials on the efficacy of acupuncture in treating IBS revealed that acupuncture significantly improves the severity of IBS symptoms (especially abdominal pain) while at the same time improving patient quality of life without any adverse effects (Yang et al., 2022a). Although acupuncture and moxibustion can regulate central and peripheral pathways, their mechanism of analgesia remains elusive. Therefore, this systematic review aims to comprehensively summarize the existing mechanism through which acupuncture and moxibustion exert an analgesic effect on IBS animal models. In other words, we investigate the effect of acupuncture stimulation on signals moving to the Central Nervous System (CNS) and affecting the peripheral pain formation process through central integration. We found the upstream and downstream pathways of the acupuncture anti-Visceral Hypersensitivity (VH) process at hypothalamic-pituitary-adrenal (HPA) axis-mediated central and peripheral levels of neurons, glial cells, and neuro-immune circuits. Our findings provide a novel approach to adopting acupuncture in IBS treatment and future research on its mechanistic role in anti-VH roles.

## 2. Methods

### 2.1. Search strategy

**FIGURE 1:**
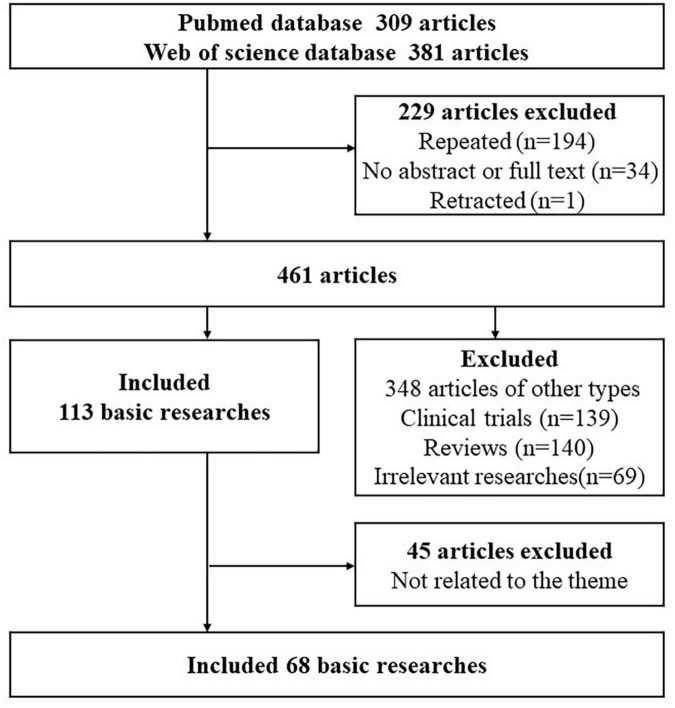
A flowchart of this search process.

To search for published studies in the PubMed and Web of Science databases, the search period was set between January 2003 and February 2023, Using [” irritable bowel syndrome “ or ” irritable colon “or” mucinous colitis “], and [” acupuncture therapy “ or ” acupuncture “ or ” meridians “ or ” moxibustion “ or ” transcutaneous electrical acupoint stimulation “ or ” electroacupuncture “ or ” auricular acupuncture “ or ” laser needle “ or ” dry needle “ or ” scalp acupuncture “ or ” fire needle “ or ” plum-blossom needle “] as keywords, setting the language only in English and Chinese. A total of 690 articles were retrieved. Before full-text evaluation, we first excluded 229 articles that were duplicated, lacked abstracts, or were withdrawn, before eliminating 139 clinical trial articles, 140 review articles, and 69 articles unrelated to the research content of this article. In total, 113 basic experimental articles remained. After careful reading and analysis of 113 basic research articles, 45 pieces of literature that could not obtain full text and those unrelated to the topic of “hyperalgesia” were excluded, and 68 basic research papers consistent with the topic were finally included. Figure 1 shows the retrieval flow chart.

### 2.2. Data extraction

We used pre-set criteria to extract data from 68 basic works of literature and analyzed the peripheral and central mechanisms of acupuncture to relieve pain in IBS by summarizing the intervention methods and specific parameters of acupuncture, as well as the effects and mechanism indicators related to acupuncture relieving pain in IBS. This process was carried out simultaneously by two researchers. Cross-comparison was performed after extraction (Table 1).

**TABLE 1:** The efficacy and neural mechanism of acupuncture in relieving visceral hypersensitivity in irritable bowel syndrome.

References	Intervention	Acupoints	Acupoint stimulation parameters	Efficacy indicators	Mechanism indicators
Yang et al., 2022b	EA	ST25, ST37	2 Hz/10 Hz, 20 min, 7 days	AWR score↓, Fecal water content↓, OT%↑	Colon: NGF↓, TrkA↓, TRPV1↓
Li et al., 2018	EA	ST25, BL25	0.1–0.3 mA, 2/100 Hz, 20 min, 5 times (once every other day)	Bristol Score↓, AWR Score↓	Colon: C-kit↓, TRPV1↓
Chen Y. et al., 2021	ACU	ST25, ST36, SP6, LR3	15 min, 14 days	PTP↑, Diarrhea index↓	DRG: Pirt↓, colon: TRPV1↓
Sun et al., 2021	GPM, MM, LM	ST25	GPM: 30 min (18 mm in diameter and 3 mm in thickness) MM: 30 min, 7 days LM: 125 mW, 2.5 mm, 30 min, 7 days	Loose stool rate↓ (GPM≈MM > LM) Bristol stool scale↓ (GPM≈MM > LM)	Acupoint: TRPV1↑; colon: TRPV1↓
Liu et al., 2012	ACU	ST36, LR3	ST36: twist frequency 120 times/min, LR3: twist frequency 60 times/min; 30 min, 14 days	Body weight↑, Food intake↑, Diarrhea index↓	Plasma: VIP↓; ileum: SS↓, SP↓, VIP↓
Xu et al., 2018	EA	ST25, ST37	2/15 Hz, 15 min, first 5 days in a week, 15 min (2 courses)	Threshold (abdomen, body lifting)↑	Colon: PAR2↓, TPSP↓, CGRP↓, SP↓
Deng et al., 2018	EA	ST25, ST37	0.2–0.3 mA, 50/100 Hz, 30 min, 14 days	Body weight↑, CRD capacity threshold↑, AWR score↓	Colon: MC↓, Try↓, PAR-2↓, TRPV1↓, SP↓, CGRP↓
Zhao et al., 2022	EA	ST25, ST36, LR3	1.5 mA, 2–15 Hz, 20 min, 14 days	AWR score↓	Colon: SP↓, BDNF↓, TrkB↓, GFAP and TrkB (colocalized in EGCs)↓, PLC↓, GFAP and BDNF (colocalized) ↓, Ca^2+^↓
Chen C. et al., 2021	MA	ST37	Twist every 5 min for 30 s	AWR Score↓	Colon: TRPV1↓, NGF↓, TrkA↓, PI3K/p-Akt↓
Bao et al., 2019	MM	ST36, ST25	2–3 cm above, 10 min, 7 days	AWR score↓, DNA abundances: (lactobacillus, bifidobacterium, faecalibacterium prausnitzii)↑, escherichia coli↓	Colon: NLRP6 ↑, ASC ↓, caspase-1↓, IL-1b↓, IL-18↓, RELM-b↓, MPO activities↓, ITLN1↑; serum: CRP↓
Song et al., 2020b	EA	ST25, ST36	0.5–1 mA; 2/5 Hz, 30 min, 10 days	Pain threshold↑, α diversity↑	Colon: IL-8↓
Song et al., 2020a	EA	ST25, ST36	0.5 mA, 2/15 Hz, 15 min, 7 days	AWR score↓	Colon: NLRP6↑, ASC↑, caspase 1↑, MUC2 ↑, E-cadherin ↑
Zhou et al., 2009	SM	ST25	15 min, 7 days	AWR score (20 mmHg)↓	Colon: 5-HT↓
Zhu et al., 2018	EA	ST25, CV12	1.5 mA, 2/15 Hz, 30 min, 6 days (2 occasions, 2 days intervals)	AWR score↓, PTP↑, VMR↓	Colon: EC cells↓, TPH↓, 5-HT↓, 5-HIAA↑
Zheng et al., 2022	BM	ST25, ST36, RN12	2 h/time, 1 time/week, 3 weeks	AWR score↓, EMG↓ (60, 80 mmHg)	Colon: 5-HT↓, 5-HT3R↓
Qin et al., 2020	EA	ST36, LI4	0.1–0.3 mA, 2/100 Hz, 20 min (once every other day), 5 days	The AWR incubation period↑, AWR contraction wave↓, bristol score↓	Colonic mucosa layer: 5-HT3AR↓; colonic muscular layer: 5-HT3AR↓
Liu et al., 2009	ACU	ST25, ST37	1 mA, 2/100 Hz, 20 min, 7 days	AWR score↓	Colon: 5-HT↓, 5-HT3R↓, 5-HT4R↑
Tian et al., 2006	EA	ST36, SP6	0.3 mA, 2 Hz, 30 min	PTP score↑, AWR score↓	5-HT↑, 5-HT4A↑; Colon: SERT↑
Zhao et al., 2017	EA, MOX	ST37	EA 1 mA group: 1 mA, 2 Hz, 10 min, 7 days EA 3 mA group: 3 mA, 2 Hz, 10 min, 7 days MOX 43°C group: use surface thermometer to confirm temperature (43± 1°C); 10 min, 7 days MOX 46°C group; use surface thermometer to confirm temperature (46 ± 1°C); 10 min, 7 days	Degree of neuronal activation↑, AWR score↓, MC↓ (MOX 46↑ group and MOX 43↑ group)	Colon: 5-HT↓, 5-HT3R↓, 5-HT4R↓
Qi et al., 2019	MM, EA	ST25, ST37	MOX Group: 10 min, 7 days, 4 mm, 2–3 cm above EA Group: 1 mA, 2/100 Hz, 20 min, 7 days	AWR score↓	Colon and SC: GDNF↓, GFRα3↓
Xu et al., 2009	EA	ST36	1 mA, 2/100 Hz, 40 min, 5 days	Colon: DRG neurons↓, VMR↓	
Wang et al., 2019	EA	ST36, ST37	EA: 1 mA, 2 Hz, 30 min, 7/14 days	DAI score↓, mechanical and thermal PWL↓, VMR reflex↓, CMDI score↓, TDI score↓	DRG: TH↓, CGRP-IR neurons↓
Tang et al., 2022	ACU	ST25, BL25	0.1–0.3 mA, 2/100 Hz, 20 min, 5 days (once every other day)	AWR score↓, stool consistency score↓, diarrhea index↓	MC↓; serum: MCP-1↓, IL-6↓, TNF-α↓, IL-1b↓, RANTES↓, CXCR3↓; colon and DRG: 5-HT↓, CGRP↓, SP↓, P2X4↓, OX42↓, BDNF↓ IRF-5↓; DRG: c-for↓
Qi and Li, 2012b	EA	ST36, ST37	Intensity: induce rats to shake; 5/100 Hz, 30 min (1, 3, 5, 7 days)	AWR score↓	NECK, NP, SDH: c-fos↓
Qi and Li, 2012a	EA	ST36, ST37	5/100 Hz, 30 min, 8 days	PTP score↑, AWR score↑	RVM: c-fos↓, NR1↓
Wu et al., 2010	EA	ST36	3 mA, 10 Hz, 20 min, 3 days	PTP score↑, AUC of EMG↑	DRN and SDH of SC: 5-HT↓; DRN, SDH of SC and colon: Fos↓
Wang L. D. et al., 2016	MOX	ST25, ST37	10 min, 7 days	AWR score↓	SC: NR1 ↑, NR2B ↑; SDH: PKCε↓; Immunohistochemistry: NR1↓, NR2B↓, PKCε↓
Liu et al., 2017	EA	ST36, ST37	1 mA, 5/25 Hz, 30 min, 7 days	AWR score↓	SDH, NP, NECK, and central canal regions (L6-S2 and T13-L2): NR2B↓
Tan et al., 2019	EA	DU29, PC6, ST36, ST25	0.1–0.3 mA, 2/100 Hz, 20 min, 5 days (once every other day)	AWR score↓, horizontal movement (DU29, PC6, ST36, ST25 group)↑ Vertical movement (ST36, ST25 group)↑	ACC: NR1↓, NR2A↓, NR2B↓; Colon: NR1↓, NR2A↓, NR2B↓
Qi et al., 2013	WM	ST37, ST25	10 min, 7 days	AWR score↓, dynorphin-immunoreactions↑	SDH: PPD↑, KOR↑, OFQ↑, spinal cord: OFQ↑
Yi et al., 2012	SM	ST25, ST37	10 min, 7 days	AWR score↓	SC: enkephalin↑
Zhao M. et al., 2020	EA	ST25, ST37	2 mA, 2/100 Hz, 20 min, 7 days	AWR score↓	MT: GFAP↓; ACC: GFAP↓
Zhou et al., 2014	MM	ST25	10 min, 7 days	AWR score	Colon and SC: PK↓, PKR↓
Chen et al., 2019	EA	ST25, ST36, SP6, LR3	1 mA, 2/15 Hz, 15 min, 14 days	Mean optical density of ZO-1↑, positive expressions of ZO-1↑, OT%↑, SP%↑	Hypothalamus: CRF↓, CRFR1↓; gastrointestinal mucosa: CRFR1↓ MC↓; the number of cells double-stained for CRFR1 and MC↓
Zhu et al., 2012	EA	ST36, LI4	0.1–0.2 mA, 2/100 Hz, 20 min, 7 days	AWR score↓	Hypothalamic: NPY↑; plasm and colon: SS↓
Mengzhu et al., 2022	EA	ST25, ST36	0.5 mA, 2/15 Hz, 15 min, 7 days	Chao 1 and simpson index↑, AWR score↓, firmicutes/bacteroidetes ratio↓	CRF expression↓, abundances of candidatus saccharimonas, dubosiella and lactobacillus↑
Zhou et al., 2011	SM	ST25, ST37	30 min (each acupoint 15 min), 7 days	AWR score↓	Hypothalamic: CRH↓
Huang et al., 2019	TENS	upper chest and back on the left or right side	Carrier frequency: 5-k Hz; 1/10 Hz (up–down sweep), 6 mA, 1 h, 7 days	EMG↓	
Zhang et al., 2022	EA	ST25	1 mA, 2/15 Hz, 20 min, 14 days	AWR score↓, AUC of EMG↓, slow-wave morphology↓	
Weng et al., 2022	EA	ST36, SP6	2 mA, 2 Hz, 15 min, 14 days	AWR score↓	SC: NMDA-NR1↓, NR2B↓, P2X7 receptor↓, GFAP↓
Wang et al., 2018	MOX, EA	ST25, ST37	2 mA, 2/10 Hz, 20 min, 7 days	AWR score↓	Firmicutes, lactobacillus, and clostridium XIVa↑; microbial composition: bacteroidetes↓, prevotella, bacteroides, and clostridium XI↓
Yoshimoto et al., 2012	TENS		3 V, 10 Hz, 0.5 ms (once every other day)	Colonic transit↓	PVN: CRF-immunoreactive cells↓, OXT-immunoreactive cells↑
Wu et al., 2008	EA	ST25	2/50 Hz, 20 min, 7 days	Pressure threshold level↑	Colon: MC↓, c-fos positive cell↓, SP↓, SPR↓, VIP↓, VIPR↓
Yang et al., 2019	EA	ST36	1 mA, 5/100 Hz, 30 min, 5 days	AUC of VMR↓, MC↓	Colon: TLR4↓, MCT↓;IL-8 ↓, IL-1b↓
Zhang et al., 2020	EA	ST25, ST37	1 mA, 2/100 Hz, 30 min, 7 days	AWR score↓, the excitability of colonic sensory neurons↓	APs propagation↓; myenteric plexus: P2X3↓, DRG: P2X3↓
Guo et al., 2013	EA	ST37, ST25	2 mA, 2/100 Hz, 20 min, 7 days	AWR score↓	Colon: P2X4 receptor immunoreactivity↓; SC: P2X4 receptor immunoreactivity↓
Weng et al., 2013	EA	ST37, ST25	2 mA; 2–100 Hz, 20 min, 7 days	AWR score↓	DRG: P2X2R↓, P2X3R↓
Liu H. et al., 2015	EA	ST37	2/100 Hz, 20 min, 7 days	AWR score↓	Colon: CRH↓, SC, and Hypothalamus: CRH↓
Ma et al., 2009	EA	ST25, ST37	2/50 Hz, 15 min, 7 days	PTP score↑	Hypothalamus: CRH↓, Colon: MC↓, SP↓, SPR↓
Liu S. et al., 2015	HSM	BL25	40 min, 8 days, height: 2 cm	AWR score↓	DRG: P2X7↓, co-expression: (P2X7 and GFAP)↓
Zhang et al., 2017	MOX	DU4	40 min, 14 days	Body weight↑, small intestinal propulsion rate↑, AWR score↓	Serum: CRH↓, ACTH↓, CORT↓
Chu et al., 2011	EA	ST36	1 mA, 5/100 Hz, 30 min, 5 days	Discharges of EMG↓	Colon: 5-HT3R↓
Lv et al., 2019	EA	ST36, ST37	1 mA, 2 Hz, 30 min, 7 days	AUC of EMG↓ hindpaw withdraw latency↑, the threshold of CFEFP↑	Spinal: LTP↓
Wu et al., 2009	EA	ST37	2/50 Hz, 20 min, 7 days	AWR score↓	Hypothalamic: CRH↓
Cui et al., 2005	EA	ST37, ST36	1 mA, 4/100 Hz, 30 min, 13 days	AWR score↓, EMG↓	
Pei et al., 2018	EA	ST25	0.5–1 mA, 2/15 Hz, 30 min, 14 days	AWR score↓	Colon: MC↓, tryptase↓, SP↓
Zhao J. et al., 2020	EA	ST36, ST37	1 mA, 2/100 Hz, 30 min, 7 days	AWR scores↓	SC: GFAP↓, P2Y1↓, IL-6↓, TNF-α↓, IL-1b, PKC↓, pERK1/2 ↓
Ji et al., 2019	EA	ST25, BL25	0.1–0.3 mA, 2/100 Hz, 20 min (once every other day), 5 days	Intestinal motility↑	Colon: M_3_R↓, 5-HT_3A_R↓
Zhang et al., 2016	EA	ST37, BL25	0.1–0.2 mA, 2/100 Hz, 30 min, 7 days	Bristol score↑, feces water content↓	Colon: CGRP↓, SP↓
Hou et al., 2022	EA	ST36, ST25, LR3	1.5 mA, 2 Hz/15 Hz, 20 min, 14 days	AWR score↓, intestinal permeability↑	GSNO↓, colocalization of GFAP and GSNO↓, occludin↓, ZO-1↓
Zhou et al., 2012	EA	ST36, BL43	1 mA; 2/100 Hz, pulse width: 0.1 ms, 30 min	AWR score↓, AUC of EMG↓	

↑, upregulated by intervention; ↓, downregulated by intervention. EA, electroacupuncture; ST25, *Tianshu*; ST37, *Shangjuxu*; AWR, abdominal withdrawal reflex; OT, open arm time; NGF, nerve growth factor; TrkA, tyrosine kinase receptor A; TRPV1, transient receptor potential vanilloid 1; BL25, *Dachangshu*; ACU, acupuncture; ST36, *Zusanli*; SP6, *Sanyingjiao*; LR3, *Taichong*; PTP, pain threshold pressure; DRG, dorsal root ganglion; GPM, ginger-partitioned moxibustion; MM, mild moxibustion; LM, laser moxibustion; SS, somatostatin; VIP, vasoactive intestinal peptide; SP, substance P; PAR2, protease-activated receptor 2; TPSP, tryptase; CGRP, calcitonin gene related peptide; MC, mast cell; Try, tryptase; PAR-2, protease-activated receptor-2; BDNF, brain-derived neurotrophic factor; TrkB, tyrosine kinase receptor B; GFAP, glial fibrillary acidic protein; MA, manual acupuncture; PI3K, phosphatidylinositol 3-kinase; p-Akt, phosphorylation-Akt; EGCs, enteric glial cells; PLC, phospholipase C; NLRP6, NOD-like receptor family pyrin domain containing 6, IL-1β, interleukin-1β; IL-18, interleukin-18; RELMβ, resistance-like molecule β; ITLN1, intestine lectin 1; MPO, myeloperoxidase; CRP, C-reactive protein; IL-8, interleukin-8; MUC2, mucin-2; SM, suspended moxibustion; 5-HT, 5-hydroxytryptamine; CV12, *Zhongwan*; VMR, visceral motor response; enterochromaffin cells; TPH, tryptophan hydroxylase; 5-HIAA, 5-hydroxyindoleacetic acid; BM, blistering moxibustion; EMG, electromyographic; 5-HT3R, 5-hydroxytryptamine 3 receptor; 5-HT4R, 5-hydroxytryptamine 4 receptor; LI4, *Hegu*; 5-HT4A, 5-hydroxyindoleacetic receptor 4A; SERT, serotonin transporter; SC, spinal cord; GDNF, glial cell line-derived neurotrophic factor; GFRα3, GDNF family receptor α3; DAI, disease activity index; CMDI, colon mucosal damage index; TDI, tissue damage index; TH, tyrosine hydroxylase; CGRP, calcitonin gene-related peptide; PWL, paw withdrawal; MCP-1, monocyte chemoattractant protein-1; IL-6, interleukin-6; TNF-α, tumor necrosis factor alpha; RANTES, regulated on activation normal T cell expressed and secreted; IRF-5, interferon regulatory factor 5; NECK, neck of the dorsal horn; NP, nucleus proprius; SDH, spinal dorsal horn; RVM, rostral ventromedial medulla; AUC, area under curve; DRN, dorsal raphe nucleus; DU29, *Yintang*; PC6, *Neiguan*; ACC, anterior cingulate cortex; WM, warming moxibustion; PPD, preprodynorphin; KOR, κ receptor; OFQ, orphanin-FQ; MT, medial thalamus; PK2, prokineticin2; PKR2, prokineticin2 receptor; ZO-1, zonula occludens-1; SP, sucrose preference; CRF, corticotropin releasing factor; CRFR1, corticotropin releasing factor receptor 1; NPY, neuropeptide; CRH, corticotropin-releasing hormone; TENS, transcutaneous electrical nerve stimulation; NMDA, *N*-methyl-D-aspartate; PVN, paraventricular nucleus, OXT, oxytocin; TLR4, toll-like receptor 4; MCT, mast cell tryptase; HSM, heat-sensitive moxibustion; ACTH, adrenocorticotrophic hormone; CORT, corticosterone; CFEFP, C-fiber-evoked field potentials; LTP, long-term potentiation; PKC, protein kinase C; M_3_R, M3 muscarinic acetylcholine receptor, BL43, *Gaohuang*.

## 3. Results

### 3.1. Peripheral analgesia mechanism of acupuncture for relieving VH

#### 3.1.1. Acupuncture inhibits local nerve cell integration of pain signals by regulating the intestinal neuro-immune network

##### 3.1.1.1. TRPV1 pathway

The Transient Receptor Potential Vanilloid 1 (TRPV1), an ion channel expressed on sensory neurons, promotes neuronal excitement by triggering Ca^2+^ inflow (Aghazadeh Tabrizi et al., 2017). Studies indicate a close relationship between TPRV1 sensitization and expression and visceral sensation (Yang et al., 2022b). Li et al. (2018) discovered that as the colonic pain threshold increased, the average colonic TPRV1 optical density value decreased after applying electroacupuncture (EA) stimulation to *Dachangshu* (BL25) or *Tianshu* (ST25). The extent of the decrease was greater in the ST25 group than that in the BL25 group, providing a definitive reference for the coordination of Shu points and mu points in gastrointestinal diseases. Furthermore, abnormal intestinal activity, secretion of neurotransmitters related to pain perception, and intestinal sensitivity were decreased by the content of c-kit (a gene expression product of Cajal stromal cells that regulates gastrointestinal motility in the digestive tract). Chen Y. et al. (2021) proposed that EA intervention applied to four acupoints, including ST25, *Zusanli* (ST36), *Sanyinjiao* (SP6), and *Taichong* (LR3) may regulate the weakening of peripheral TRPV1 signaling and reduce phosphoinositide binding protein Pirt (an endogenous enhancer of TRPV1 function (Kim et al., 2008)) expression in DRG. Sun et al. (2021) compared the differences in the effects of ginger-partitioned moxibustion, mild moxibustion, and laser moxibustion on IBS-D rats. Unlike the control group, a higher-than-normal expression of the TRPV1 receptor was observed in the ST25 acupoint and colon tissue of the IBS group, suggesting that the acupoint could have been activated from the dormant state in the model condition. Besides, the colonic tissues exhibited pathological activation. However, the three moxibustion interventions further increased TRPV1 receptor expression in acupoint tissue but decreased the TRPV1 receptor expression in colon tissue. Consequently, research speculates that moxibustion could affect IBS-D by activating TRPV1 channels in acupoint tissue via thermal stimulation, inducing acupoint signal activation, inhibiting the activation state of colon TRPV1 ion channel, reducing intestinal hypersensitivity, regulating colon movement, and improving abdominal pain as well as diarrhea symptoms. However, whether acupuncture specifically blocks TRPV1 channel expression on the sensory nerves of colon tissue remains to be clarified. Furthermore, there is a need to further investigate whether acupuncture affects TRPV1 channel activity and decreased expression.

Following stimulation by inflammatory factors, sensory neurons located in the intermuscular or submucosal plexus of the colon depolarize when IBS occurs. The specific TRPV1 ion channels on the cell membrane are opened, and neuropeptides [including Calcitonin Gene-Related Peptide (CGRP) and Substance P (SP)] originally stored in the vesicles of these neurons, are released. Nine of the selected studies were centered on acupuncture effects on gene and protein levels of local CGRP and SP neuropeptides in the colon. The acupoints selected were mostly ST25 and *Shangjuxu* (ST37). The sparse wave EA intervention was applied in all studies except for one on colon SP content that selected hand acupuncture intervention (Liu et al., 2012). For instance, Xu et al. (2018) discovered that the mRNA and protein levels of SP and CGRP in the colon decreased after ST25 and ST37 stimulation using 2/15 Hz EA. Another study that selected the same acupoint and executed at the same protein level but with a higher density wave parameter (50–100 Hz) not only showed similar results as the former but also found a decreased protein content of Protease-Activated Receptor 2 (PAR2) and TRPV1 in the colon (Deng et al., 2018). The PAR2 is highly expressed in the rectal mucosa, and the two drug-induced IBD mouse models, it is translocated from the outer colonic basement plasma membrane to the early endosomes of colonic cells. By increasing paracellular permeability, releasing pro-inflammatory substances, and sensitizing colonic injury receptors, PAR2 endocytosis induces inflammation and pain (Latorre et al., 2022). Furthermore, Liu et al. (2012) and Zhao et al. (2022) discovered that simultaneous intervention with ST36 and LR3 could diminish the SP content in the colon.

Many inflammatory factors, bradykinin, Nerve Growth Factor (NGF), etc., can directly or indirectly sensitize or activate TPRV1. The PAR2, Tyrosine kinase-type receptor (Trk), and multiple signal transduction pathways are involved in the process (Ji et al., 2002; Kim et al., 2014). One emerging target for treating intractable pain is the NGF and its receptor Tyrosine kinase-type receptor A (TrkA), which has a high affinity for NGF (Hirose et al., 2016). Additionally, the combination of NGF and TrkA might sensitize and improve TRPV1 expression (Donnerer et al., 2005). Electroacupuncture in ST25 and ST37 can downregulate NGF and TrkA expression in colon tissue of IBS-D model rats (Yang et al., 2022b), providing a new avenue for TRPV1 inhibition. In a study involving manual acupuncture treatment of ST37, the NGF-PI3K-TRPV1 axis is presumed to exert a significant influence on colonic hyperalgesia (Chen C. et al., 2021). By initiating and regulating paracrine signals, various types of immune/inflammatory cells present in the intestinal mucosa, laminae propria, and smooth muscle, including leukocytes, lymphocytes, macrophages, dendritic cells, and mast cells (MCs), can influence the Enteric Nervous System (ENS) and vagus nerve fibers, thus producing visceral pain (Grundy et al., 2006). These cells produce essential pro-inflammatory factors including Interleukin (IL)-8 and IL-18. The IL-18 can aggravate the inflammatory environment by further inducing IL-1β production by Th1 and natural killer cells (Grover, 2014). Low-grade inflammatory nerves in the colonic mucosa are involved in the frequency and severity of perceived abdominal pain in non-specific IBS (Barbara et al., 2004). Bao et al. (2019) discovered that mild moxibustion of ST36 and ST25 could considerably reduce the expression of IL-1β and IL-18 mRNA and protein in the intestinal tissues of Post-Inflammatory (PI)-IBS rats, hence suppressing inflammation, which was superior to the sham moxibustion group. Additionally, it could upregulate the NOD-Like Receptor Protein 6 Inflammasome (NLRP6) gene and protein levels in the colon. As a negative regulator, NLRP6 can suppress the inflammatory signaling pathway by reducing IL-1β and IL-18 expression. Furthermore, during EA stimulation at the same acupoints, Song et al. (2020a,b) observed upregulated NLRP6 expression and downregulated IL-18 expression in the colon. At the same time, based on intestinal immunofluorescence results, the fluorescence NPLR6 signals highly overlapped with those of adhesion connexin E-cadherin in intestinal epithelial cells and cell junctions, suggesting a restoration of intestinal barrier function and reduction of inflammatory degree, respectively. By activating MCs and macrophages, intestinal inflammation can trigger IL-1β secretion into the inflammatory environment. In turn, IL-1β stimulates the formation of nociceptor-sensitive substances including Prostaglandin E2 (PGE2) and NGF, as well as rapid and direct activation of nociceptors to cause action potentials and hyperalgesia. The IL-1β sensitizes TRPV1 via its corresponding receptor, dependent on the downstream Protein Kinase C (PKC) signaling pathway, and induces hyperalgesia (Binshtok et al., 2008). The above studies indicate that acupuncture may inhibit the upload of pain signals in the TRPV1 channel of intestinal neurons to achieve analgesia by improving the intestinal inflammatory environment. This acupuncture inhibitory effect may be achieved by improving NLRP6 expression levels, blocking the release of inflammatory factors, or NGF/TrkA signaling pathway, thus inhibiting TRPV1, i.e., TRPV1 may be a target for improving intestinal inflammation in IBS patients through acupuncture.

Apart from establishing an inflammatory environment, activated colon MCs may also participate in TRPV1 channel activation in the ENS via other pathways. Tryptase (TPSP), a ligand that activates PAR2 on sensory neurons in the colon, is released when MCs degranulate upon stimulation (Xu et al., 2018). Studies suggest that PAR2 can activate the TRPV1 receptor and upregulate TRPV1 expression, via co-localization on the primary afferent nerve, thereby sensitizing the TRPV1 receptor and causing its phosphorylation, improving SP and CGRP release, and further mediating pain hypersensitivity (Deng et al., 2018). By down-regulating the mRNA and protein expression of PAR2 and TPSP (the key mediator of PAR2) in the colon, the combination of specific acupoints, including ST25 and ST37, or ST36 and ST37, may block PAR2 activation (Deng et al., 2018; Xu et al., 2018), suggesting that TPSP/PAR2 could be another upstream mechanism of stimulating TRPV1 ion channel to relieve VH using EA. Several studies indicate that acupuncture can prevent the activation of MCs in the intestine. For example, through toluidine blue staining, five EA studies and one on moxibustion discovered decreased colon MC count after the intervention. The acupoints involved were restricted to ST25, ST37, and ST36, with most studies using the sparse wave intervention mode. These findings support the evidence that acupuncture participates in the TRPV1 pathway by inhibiting MC activation. Therefore, considering that it blocks MC degranulation and prevents the opening of TRPV1 channels on the cell membranes of intestinal neurons, acupuncture may be one of the initial anti-VH stages.

##### 3.1.1.2. Serotonin pathway

Serotonin/5-Hydroxytryptamine (5-HT), a key gastrointestinal signaling molecule, stimulates intestinal primary afferent neurons, initiates peristalsis and secretory reflexes, and transmits information to the CNS (Gershon and Tack, 2007). Since 5-HT in the ENS is expected to increase in response to visceral pain (McLean et al., 2007), it is often considered a visceral pain indicator (Zhou et al., 2009). Among all the studies included in this review, the experimental results of 4 studies on EA and 5 studies on moxibustion revealed that acupuncture can decrease intestinal 5-HT expression in IBS rats. Zhu et al. (2018), for instance, discovered that Trinitrobenzene Sulfonic (TNBS)-induced colon EC cells of IBS rats exhibit proliferation, and abnormally high expression of 5-HT, and Tryptophan Hydroxylase (TPH). This process was significantly reversed by EA stimulation of ST25 and Zhongwan (CV12). Additionally, the process significantly increased 5-Hydroxyindoleacetic acid (the main metabolite of 5-HT) concentration. In other words, EA stimulation can reduce and promote production and rapid metabolism of 5-HT, respectively (Zhu et al., 2018).

Recent research reports indicate that specific 5-HT subtypes and receptors determine whether 5-HT can promote or inhibit neuropathic pain (Liu et al., 2020). Simultaneously, by binding with different receptors, 5-HT can exert anti-inflammatory or pro-inflammatory effects on the gut (Liu et al., 2021). The 5-HT in the intestines primarily stimulates 5-HT3 and 5-HT4 receptors on the primary afferent neurons in the gut and vagus nerve fibers to regulate sensory and motor responses. The findings revealed that EA, moxibustion, and 5-HT3 receptor antagonist therapy suppressed and reduced VH in IBS patients via a similar mechanism. The process of incoming signal transmission was hindered by blocking the binding of 5-HT and 5-HT3 subtype receptors, lowering VH and rectal sensitivity in IBS patients. After applying natural moxibustion to CV12, ST25, and ST36 acupoints, Zheng et al. (2022) observed decreased average positive area rate of 5-HT3 receptor protein in colon tissue. On the other hand, after only applying EA to the ST36 acupoint, Chu et al. observed a decrease in 5-HT3 receptor mRNA in the colon (Chu et al., 2011). Furthermore, a decrease in the positive expression of the 5-HT3A receptor in the colonic mucosal layer of the IBS model was discovered in another study on blistering moxibustion (Qin et al., 2020). On the other hand, the 5-HT-specific stimulation of 5-HT4 receptors on colonic dermal sensory neurons suppresses inflammatory responses (Liu et al., 2021). Using immunohistochemical technology, two studies were conducted on specific acupoints (ST36 combined with CV12 and ST25 combined with ST37), and the results revealed that EA could slow down VH and increase 5-HT4 receptor concentration in the colon (Tian et al., 2006; Liu et al., 2009). These findings indicate that by promoting 5-HT4 receptor expression, EA could potentially play an anti-inflammatory role, hence inhibiting VH occurrence. However, the extent to which varied acupoint compatibility affects the 5-HT4 receptor warrants additional investigation. Additionally, a study on ST36 revealed a decreasing trend in the expression of the 5-HT4 receptor, which contradicts the findings of other researchers (Zhao et al., 2017). Another study revealed that acupuncture targeting ST25 and ST37 acupoints only increased the 5-HT4 receptor concentration in the colon but had no effect on the concentration of the 5-HT3 receptor (Liu et al., 2009). Consequently, there is also a need to further explore the influence of varied acupoint selection and intervention strategies on the differences in the action targets of the colon serotonin pathway.

Furthermore, Epac1/Piezo2, as the upstream of 5-HT, participates in the release of serotonin by EC cells. Piezo2 is involved in gastrointestinal sensory regulation, and its expression level in gastrointestinal epithelial EC cells and gastroenteric-related Dorsal Root Ganglion (DRG) is relatively higher (Bai et al., 2017). One study demonstrated that EA stimulation on ST36 and ST37 could reduce AWR scores as well as the mRNA and protein expressions of Epac1, Piezo2, 5-HT3, and 5-HT3 receptors in distal colonic tissue, which was better than the IBS group and the sham EA group (Guo et al., 2022). This finding supports the conclusion that through the Epac1/Piezo2 pathway, EA may block the expression of the 5-HT3 receptor on intestinal sensory neurons, suppressing the transmission of pain signals between neurons to exert an anti-VH effect.

##### 3.1.1.3. Enteric glial cells

Under healthy conditions, intestinal Enteric Glial Cells (EGCs) provide support and nutrition to nerve cells, but under IBS, they could trigger chronic intestinal inflammation through specific secretion of neurotrophic factors when over-activated (Kim and Camilleri, 2000). Increased intestinal BDNF binds to TrkB receptors on the surface of intestinal submucosal EGC, and activates the PLC-IP3 signaling pathway, causing a sharp increase in Ca^2+^ levels in EGCs and EGC over-activation (Leal et al., 2014; Liang et al., 2016; Wang P. et al., 2016). Contrary to the effects observed in the EA combined with the TrkB agonist group (Progatzky et al., 2021), the EA stimulation of ST25, ST36, and LR3 in IBS-D rats can reduce BDNF and TrkB genes in the colon, promote the recovery of abnormal EGCs morphology, and prevent over-activation-induced intestinal immune disorders. Another experiment demonstrated similar results. After co-staining BDNF and TrkB with Glial Fibrillary Acidic Protein (GFAP), an active marker of EGCs, conspicuous co-localization results in the model group and decreased co-localization fluorescence intensity after EA was observed (Zhao et al., 2022). These findings demonstrate that by inhibiting the BDNF/TrkB signaling pathway, acupuncture may reduce the over-activation of intestinal EGCs and alleviate IBS-D in IBS rats. Besides reducing the mRNA and protein expression of Glial-cell-line-Derived Neurotrophic Factor (GDNF) and its intestinal glial cell-secreted GDNF Family Receptor alpha 3 (GFRα3), moxibustion or EA can improve the intestinal inflammatory environment and promote the recovery of intestinal mucosa (Qi et al., 2019). Since EGCs can support and nourish the ENS while activating and maintaining the inflammatory environment (Grubišić and Gulbransen, 2017), intestinal EGCs have become important in VH development. Therefore, considering that EGCs also secrete neuropeptides including SP and CGRP, we hypothesize that EGCs in the gut may directly mediate the inhibitory effect of acupuncture on neighboring nerve cell excitation.

#### 3.1.2. Acupuncture prevents pain signals from reaching higher levels by interfering with DRG neurons

The Dorsal Root Ganglion (DRG), is a crucial structure in sensory transduction and regulation, including pain transmission. Stimulating DRG to regulate the transmission of pain signals to the CNS as a “relay station” connecting peripheral and central signal transmission is considered a novel nerve stimulation approach for chronic refractory pain treatment (Deer et al., 2013). Piezo2 is expressed in over 45% of DRG neurons (Roh et al., 2020). As per several studies, Piezo2 mediates sensitized mechanical pain caused by inflammation and nerve injury. Consequently, the targeted suppression of the Piezo2 protein channel in sensory neurons may be an effective strategy for treating mechanical allydonia (Murthy et al., 2018). Acupuncture application to ST36 and ST37 reduced the mRNA and protein expression of Epac1 and Piezo2 in L5-S2 DRG neurons. Additional investigations into the membrane electrical properties of colon-related DRG neurons revealed that EA significantly downregulated and upregulated the Resting Membrane Potential (RMP) of colonic DRG neurons and basal rheological properties of sensitized neurons, respectively, and inhibited the number of action potential propagating and excitability of sensitized neurons (Guo et al., 2022). Elsewhere, we found that within the DRG, EA can regulate DRG neurons’ excitability by significantly suppressing the expression level of P2X3 receptor protein and mRNA in P2X3 positive neurons associated with injury information (Zhang et al., 2020). Furthermore, in the IBS model group, significant depolarization and increase in action potential and discharge were discovered on DRG neurons in the thoracolumbar spine (including T13, L1, and L2) and lumbosacral (including L6, S1, and S2). The EA stimulation of ST36 reduced the excitability of DRG neurons in the colon of CVH rats; however, this effect was reversed after peripheral opioid antagonists were used. After using the opioid agonists alone, the action potential number of lumbosacral DRG neurons decreased (Xu et al., 2009), suggesting that the analgesic effect of EA via DRG nerves is predominantly mediated by peripheral opioids.

In a specific study, the observation of co-expression between tyrosine hydroxylase (TH) labeled sympathetic nervous system fibers and CGRP positive neurons within L6 DRG provided definitive evidence. This evidence established that, in the model group, the sympathetic nerve fibers labeled with TH exhibited migration toward the cell layer of DRG sensory neurons. However, EA reversed this phenomenon and blocked TH expression in the dorsal root ganglia (Wang et al., 2019). In the context of colitis rats, ST36 and ST37 could inhibit the sympathetic fiber bud of L6 DRG sensory neurons. Additionally, EA reduces the content of CGRP and SP in DRG neurons (Tang et al., 2022). However, further studies are required on the upstream and downstream pathways of CGRP and SP in DRG neurons regulated by acupuncture.

On the one hand, TRPV1 expression on intestinal sensory neurons is inhibited by the summing of the peripheral mechanism of acupuncture intervention in intestinal VH. By reducing the IL-8, IL-18, and IL-1β levels, acupuncture can improve the inflammatory environment and suppress TRPV1 activation. Additionally, acupuncture can reduce TPSP secretion by inhibiting MC activity, hence restricting PAR2 expression on intestinal nerve cells. The PAR2/TPSP, an upstream of TRPV1, further reduces nerve impulse production, and NGF/TrkA, as another upstream factor, may also participate in this process. On the other hand, acupuncture can suppress neurotransmission and chronic inflammation by targeting 5-HT3 receptors on intestinal nerve cells, promoting 5-HT4 expression, and reducing 5-HT content in the intestine. The Epac1/Piezo2 and P2X3 receptor pathways may be the primary linkages that inhibit the excitability of DRGs.

### 3.2. The central analgesic mechanism on acupuncture relief of VH

According to several studies, analgesia at the CNS level is primarily due to different levels of integration between the afferent signals from the pain area and the impulses from acupoints in the dorsal horn of the spinal cord and the medial thalamus.

#### 3.2.1. Acupuncture inhibits central neuron excitation to achieve anti-VH

The spinal nerves in the thoracolumbar T13-L2 and lumbosacral L6-S2 spinal segments transfer nociceptive information from the colon and rectum to the CNS. The superficial and deep peroneal nerves are located at the ST36 acupoint and connect to the spinal cord at the L6-S2 segment (Zhao et al., 2017). The number of c-fos positive neurons in the superficial lamina propria, dorsal horn neck segment of L6-S2 segment, and the dorsal horn neck segment of T13-L2 segment of the spinal cord decreased after EA stimulation of ST36 and ST37 (Qi and Li, 2012b). A reduced count of c-fos protein and NMDA Receptor 1 (NR1) receptor-positive neurons in the rostral ventromedial nucleus of the medulla oblongata was also observed when IBS rats were subjected to EA stimulation of ST36 and ST37 (Qi and Li, 2012a). This could explain why EA causes an abnormal decline in the excitability of visceral responsive neurons in this brain area. In congruence with this finding, a previous study concluded that EA stimulates spinal dorsal horn neurons and relays acupuncture signals to the brain, activating descending inhibitory signals, thereby suppressing the expression of c-fos and NR1 in the spinal dorsal horn (Xu et al., 2010). Similarly, stimulating ST36 with EA alone significantly suppressed Fos-positive cells and 5-HT expression in the spinal cord (specifically in the dorsal raphe nucleus and shallow dorsal horn); however, similar effects were not observed with sham EA (Wu et al., 2010). Another study that solely intervened ST37 using EA or moxibustion compared the extent to which different stimulation approaches and intensities reduced VH. Consequently, wide dynamic range neurons in the dorsal horns of the spinal cord were significantly activated by relatively high current intensity (3 vs. 1 mA) and moxibustion temperature (46 vs. 43°C) (Zhao et al., 2017). By inhibiting the activation of dorsal horn neurons in the spinal cord segment corresponding to the pathological gut, both EA and moxibustion can suppress the descending pain signal transmission.

##### 3.2.1.1. NMDAR ion channels

NMDAR activation in the dorsal horn of the spinal cord is an important factor driving the development and maintenance of hyperalgesia (Urban and Gebhart, 1999). NMDAR comprises three subunits, i.e., NR1, NR2, and NR3. NR1 is the basic subunit, and each NMDA Receptor 2 (NR2) subunit has distinct NMDARs with unique functional properties (Li et al., 2009; Paoletti et al., 2013). NR1 and NR2B mRNA and protein significantly increased in the spinal dorsal horn of rats with IBS visceral pain hypersensitivity. Application of moxibustion ST25 and ST37 reversed this increase. Interestingly, the efficacy of moxibustion in alleviating visceral pain was slightly better than that of the intrathecal injection of specific NMDA receptor antagonists; this shows that moxibustion can also suppress visceral pain via other pathways (Wang L. D. et al., 2016). In contrast to the IBS model group, the ST36 and ST37 asthenia downregulated the expression level of NR2B subunit of spinal dorsal horn neurons, majorly by targeting the surface laminae (laminae I and II), nucleus propria (laminae III and IV), cervical dorsal horn (laminae V and VI) and the central canal (laminae X) at the level of thoracolumbar segment (T13-L2) and lumbosacral segment (L6-S2), which could not be achieved with sham EA therapy (Liu et al., 2017). Similar to the inhibition of TPPV1 channels after applying peripheral analgesia, acupuncture can inhibit central nervous cell excitation by targeting the VH-associated NMDAR ion channels on the cell membrane.

Anterior Cingulate Cortex (ACC) participates in the regulation of pain and pain-related mood disorders (Li et al., 2019). In ACC, two major forms of long-term potentiation (LTP) co-exist in excitatory synapses which promote the occurrence of chronic pain and pain-related mood disorders. Postsynaptic LTP induction depends on the activation of postsynaptic NMDARs (Chen Q. et al., 2021). Several studies have evaluated abdominal pain and abnormal behavior in IBS rats by comparing the stimulation of different acupoints [*Yintang* (DU29), *Neiguan* (PC6), ST36, and ST25] through EA. The findings indicate that EA significantly decreases NR2A and NR2B in the ACC region, specifically in the DU29 group. In addition, the effects of different acupoints vary, with only NR2B showing a decrease in the ST25 group (Tan et al., 2019). Based on these results, ACC mediated the modulatory effect of EA on abdominal pain in IBS rats, and this effect was influenced by the distribution of acupoints. In addition, the role of medullary subnucleus reticularis dorsalis-ACC loop in reducing visceral hypersensitivity has also been suggested (Yu et al., 2018).

##### 3.2.1.2. Opioid pathway

Opioid peptide activity in the central nervous system regulates pain transmission (Witt and Davis, 2006). Endogenous opioids in the spinal cord also mediate the analgesic effect of mild moxibustion. Mild moxibustion stimulation of ST25 and ST37 upregulates the mRNA and protein levels of dynorphin and orphanin in the spinal regions, causing partial alleviation of VH by activating the spinal dynorphin and orphanin systems. This analgesic effect can be reversed by intrathecal injection of antagonists of both therapies (Qi et al., 2013). Yi et al. (2012) employed the moxibustion method to stimulate acupoints ST25 and ST37 in an IBS model. The results revealed that the spinal enkephalin levels increased in the model group, and this effect was further amplified after moxibustion intervention, resulting in a further increase in enkephalin concentration. Acupuncture blocks pain signal transmission in the central system by secreting opioids. However, studies on the upstream and downstream mechanisms of the IBS central opioid pathway mediating the effects of acupuncture analgesia are limited. Only two studies have specifically investigated moxibustion, possibly reflecting the advancement of research on the opioid pathway. Many researchers have shifted their focus to investigating alternative analgesic pathways. Generally, the heat stimulation signal generated by moxibustion bears a resemblance to pain signals, and both signals are transmitted to the spinal cord, causing postsynaptic inhibition and pain transmission suppression. These physiological and morphological mechanisms act as the basis for the analgesic effects of moxibustion.

#### 3.2.2. Acupuncture interferes with central glial cells to achieve anti-VH

The “tripartite synapse” idea proposed by Vladimir Parpura adds glial cells to the original classical presynaptic and postsynaptic neuron cells, emphasizing its important role (Lyu et al., 2021). Activation of central glial cells is critical in VH occurrence and persistence. Previous studies have shown the effects of acupuncture on central astrocytes, exemplified by a study that revealed acupuncture-induced alterations in the ultrastructure and functionality of astrocytes in specific brain regions, including the hippocampus (Tang et al., 2019). EA at ST25 and ST37 downregulated the expression of GFAP gene and protein in the medial thalamus and ACC, however, EA reverses this phenomenon (Zhao M. et al., 2020). For IBS rats, stimulating ST36 and ST37 by EA down-regulated the expression of NMDA receptors in the spinal cord which transmit nociceptive stimuli. The astrocyte P2X7 receptor in the spinal cord regulates Ca^2+^ signals through the NMDA receptor, positively correlates with VH, and mediates the interaction between neurons and glial cells in the central nervous system. Unlike the IBS group, EA reversed the overexpression of the P2X7 receptor in the spinal dorsal horn of IBS rats, and down-regulated NMDAR, NR1 subunit, and GFAP expression. The P2X7 receptor antagonist combined with EA improved the effect of EA, whereas the application of P2X7 receptor agonist partially reversed the analgesic effect of EA, as well as suppressed the down-regulation of NR1, NR2B, and GFAP expression (Weng et al., 2022). This suggests that EA can inhibit Ca^2+^ influx and cell activation in IBS rat central astrocytes by suppressing the expression of the P2X7 receptor, thereby weakening the nerve impulse signal to prevent the occurrence of VH.

Prokinetic protein (PKs) is highly expressed in the dorsal root ganglion and nociceptor terminals. It activates the PKs receptors in the spinal dorsal horn and astrocytes, thereby promoting central sensitization and maintaining chronic and neuropathic pain (Negri and Maftei, 2018). Two studies on mild moxibustion revealed that ST25 can inhibit the protein and mRNA expression of PK1/Prokineticin receptor (PKR) 1 and PK2/PKR2 in the spinal cord of IBS rats, respectively (Zhao et al., 2012; Zhou et al., 2014). These findings suggest that acupuncture may exert its effects by suppressing the activity of central glial cells by inhibiting P2X7 and PKs pathways in the spinal cord. This mechanism could potentially alleviate central pain sensitization associated with visceral sensitivity in individuals with IBS. The aforementioned studies revealed that acupuncture intervention suppressed the activation of central sensory neurons, majorly by downregulating NMDAR expression and its subunits in the dorsal horn and ACC of the spinal cord. Therefore, we postulate that acupuncture may alleviate dorsal horn pain of the spinal cord to the brain region by inhibiting the NMDAR channel, glial P2X7 pathway, and spinal opioid peptide secretion in the dorsal horn of the spinal cord.

### 3.3. The HPA axis mechanism on acupuncture relief of VH

#### 3.3.1. Depression and anxiety induce visceral hypersensitivity in IBS

Individuals with IBS are at high risk of developing anxiety and depression (Pinto-Sanchez et al., 2017). A Korean cohort study of newly diagnosed IBD patients revealed that the cumulative incidence of depression and anxiety was 8 and 12% (Choi et al., 2019), respectively, after 6 years of follow-up. In individuals with IBS, significant associations have been observed between anxiety and depression symptoms and brain regions including the hypothalamus, amygdala, ACC, and anterior insula (Ellingson et al., 2013; Hubbard et al., 2018). These brain regions are responsible for the central regulation of chronic pain. Notably, a decreased activity in the central amygdaloid-ventrolateral mesencephalic periaqueductal gray matter pathway may promote pain perception in depression (Yin et al., 2020). Therefore, improving anxiety and depression in IBS patients may be an effective approach to interrupt the vicious cycle of stress and alleviate symptoms, and therefore should be performed throughout IBS treatment.

Acupuncture can significantly relieve symptoms of gastrointestinal disorders (FGID) including IBS (Wang et al., 2021), and it is more effective than drugs in relieving the emotions of FGID patients (Wang et al., 2022). An fMRI investigation revealed that manual acupuncture applied to ST36 effectively influenced neural activity across various tiers of the cerebro-cerebellar and limbic systems (Wang et al., 2022). Notably, the afferent signals stemming from acupuncture at LI4 and *Sibai* points were observed to converge onto the same nucleus tractus solitarius neurons, which exert a dual influence on both the digestive system and the brain (Qin et al., 2008). Moreover, it can increase the connection of the amygdala to the periaqueductal gray matter and insula of the midbrain, as well as improve nociceptive hypersensitivity in depressed states by regulating this pathway (Qin et al., 2008). Acupuncture at *Baihui* (GV20) and DU29 effectively counteract the chronic stress-induced down-regulation of BDNF mRNA and protein expression in the frontal cortex and hippocampus of rat models of depression. This restorative effect on BDNF expression contributes to the antidepressant-like effects of acupuncture by promoting neuronal regeneration and protection (Liang et al., 2012). Neuropeptide Y (NPY) is among the principal neuropeptides found within the intestinal submucosal plexus, with widespread presence in the brain as well. Acting as an anti-stress neuromodulator, NPY has demonstrated notably diminished concentrations in the cerebrospinal fluid and plasma of individuals with depression (Zhang et al., 2017). Fundamental investigations have indicated that the reduction of NPY levels in rats modeled with irritable bowel syndrome (IBS) might contribute to heightened stress responses and a cascade of inflammatory reactions, including visceral hypersensitivity. Furthermore, EA at ST36/LI4 effectively promotes the recovery of NPY content in the thalamus of rats with IBS, significantly increasing the NPY content, and alleviating the symptoms related to IBS (Zhu et al., 2012). In the superficial skin, DU29 is supplied by the supratrochlear nerve, while at the muscle level, the temporal branch of the nerve innervates it. Acupuncture performed at DU29 has the direct capability to transmit impulses to the inferior colliculus located in the middle brain and pons. Notably, clinical investigations have ascertained that the mechanism underlying the efficacy of electroacupuncture at DU29 for addressing insomnia and chronic pain could be attributed to the direct entry of nerve impulses into the brain post-acupuncture. This entry stimulates the release of neurotransmitters such as 5-HT, norepinephrine, and brain-derived neurotrophic factor (NE and BDNF), thereby instigating antidepressant and analgesic effects (Qin et al., 2008).

#### 3.3.2. Acupuncture may improve IBS stress-induced visceral hypersensitivity and emotional comorbidity through the HPA axis

The stress signals associated with IBS converge in the hypothalamus, resulting in the activation of the HPA axis. This activation increases the levels of corticotropin-releasing hormone (CRH), adrenocorticotropic hormone (ACTH), and corticosterone (CORT). Consequently, the negative feedback loop of the HPA axis becomes disrupted, exacerbating the clinical symptoms associated with IBS (Zhang et al., 2017). The findings revealed that acupuncture could treat IBS through the HPA axis and improve VH. Acupuncture at ST36 improves the working energy of the cerebral cortex and regulates the pituitary-adrenal cortex system bidirectionally, and the gastrointestinal function. EA applied at acupoints ST36 and ST35 decreases the level of CRH in the colon of individuals with IBS (Mengzhu et al., 2022). Furthermore, heat-sensitive moxibustion suppresses the expression of CRH, ACTH, and CORT in the HPA axis, subsequently improving gastrointestinal motor function (Zhang et al., 2017). The expression of CRH protein and mRNA in the colon, spinal cord, and hypothalamus is regulated by EA at ST37, which also reduces the VH of rats (Liu H. et al., 2015). Suspension moxibustion down-regulated CRH mRNA expression in the hypothalamus and significantly reduced the sensitivity of rat viscera to colorectal dilatation (Zhou et al., 2011).

The HPA axis can mediate VH-induced depression and anxiety. Acupuncture improves depression and anxiety symptoms by modulating the HPA axis. In chronic and unpredictable mild stress (CUMS) rat models, EA application decreased the hypothalamic CRH mRNA expression, downregulated plasma ACTH and CORT levels, and significantly increased hippocampal serotonin concentration and 5-HT1AR (mRNA and protein) expression. This suggests that EA can regulate the HPA axis and enhance the hippocampal 5-HT/5-HT1AR on depression in CUMS rats (Le et al., 2016). The dysregulation of the HPA axis is closely linked to the manifestation of depression, anxiety, and VH symptoms in individuals with IBS. Acupuncture has the potential to transmit signals to the brain, hence releasing neurotransmitters and hormones. This process occurs by activating the amygdala-midbrain periaqueductal gray matter pathway, as well as the midbrain, hypothalamus, and pons. Consequently, this neural modulation suppresses the expression of CRH, ACTH, and CORT in the hypothalamus, spinal cord, and colon. Through the modulation of these factors, acupuncture can potentially alleviate IBS-related depression, anxiety, and VH symptoms. The regulation of the HPA axis by acupuncture has been extensively reported, however, limited studies have demonstrated whether acupuncture by regulating the HPA axis can improve the VH induced by depression and anxiety in IBS. Previous studies have provided important insights to guide the development of acupuncture for the treatment of IBS. Besides, In a systematic review of global, population-based studies that evaluated the prevalence of IBS, the disease was more common in women (14%) than men (9%) (Lovell and Ford, 2012a). Perhaps, the greater anxiety and depression of women alter HPA axis pain processing and predispose to more severe GI tract symptoms in women. The extent to which acupuncture can improve visceral hypersensitivity in IBS patients of different genders is also worth further discussion.

## 4. Discussion

Existing studies provide strong evidence that acupuncture interventions for treating visceral hypersensitivity VH in IBS primarily use EA and moxibustion therapy. Among these methods, there is a specific emphasis on investigating the efficacy of sparse wave EA. The selection of acupoints in the treatment of VH in IBS primarily focuses on ST25, ST37, and ST36. These acupoints are commonly used individually or in combination. The most frequently used combination is ST25 and ST37, whereas ST25 is predominantly used when a single acupoint is selected. Regarding the EA parameters, sparse wave stimulation is commonly employed; however, significant variations have been noted in the choice of frequency. Studies have shown that 2∼10 Hz EA frequency effectively inhibits inflammation and neuropathic pain more than 100 Hz EA frequency (Zhang et al., 2014). The literature for this study commonly used low-frequency sparse wave stimulation ranging between 2 and 10 Hz. Furthermore, additional studies have shown that ginger moxibustion, characterized by fluctuating temperature, and mild moxibustion techniques effectively induce acupoint sensitization, achieving better treatment outcomes than laser moxibustion, which involves stable temperature application (Sun et al., 2021). Two additional studies documented noteworthy suppression of visceral hypersensitivity resulting from neuronal activity within the subnucleus reticularis dorsalis and wide dynamic range regions, employing EA at intensities ranging from 2 to 6 mA (Yu et al., 2018; Yu et al., 2019). These pieces of evidence indicate that in the investigation of the neural mechanisms underlying the relief of VH, there is a relatively concentrated focus on the selection of acupoints. However, additional research is necessary to confirm the effects of specific intervention parameters on molecular indicators.

**FIGURE 2:**
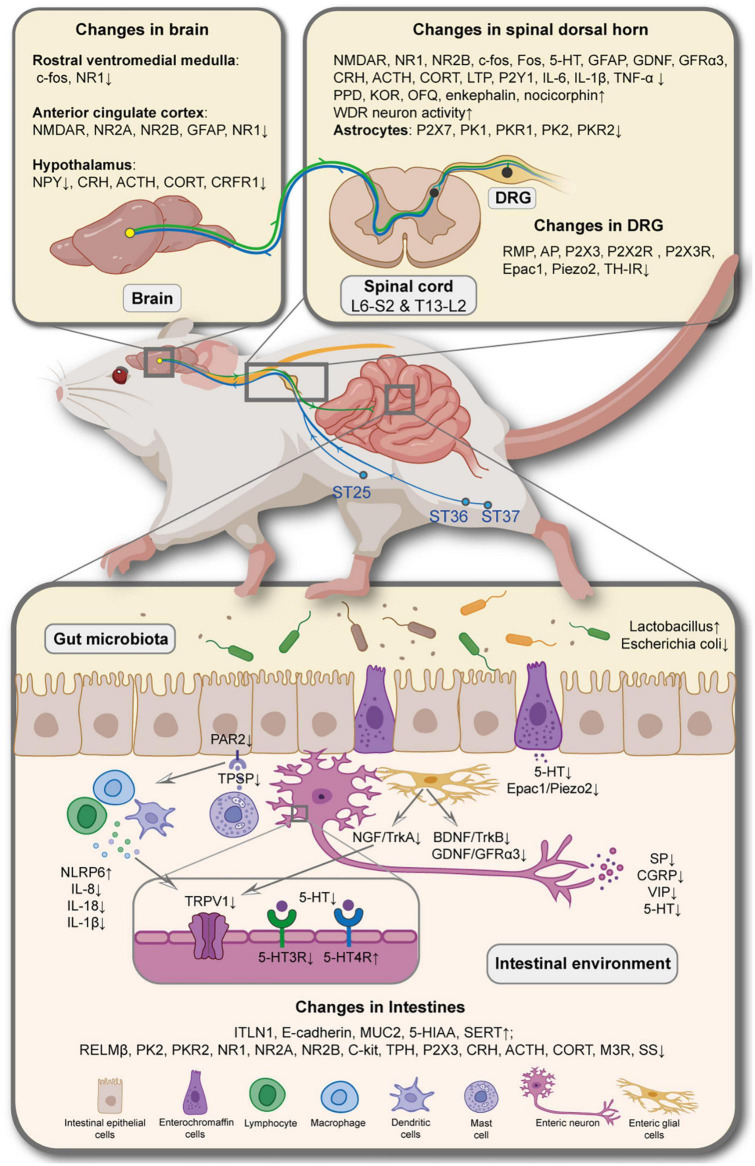
Potential neuroimmune regulatory mechanisms involved in acupuncture for relieving visceral hypersensitivity in irritable bowel syndrome. NMDAR, *N*-methyl-D-aspartate receptor; NR1, NMDA receptor 1; NR2A, NMDA receptor 2A; NR2B, NMDA receptor 2B; GFAP, glial fibrillary acidic; NPY, neuropeptide; CRH, corticotropin-releasing hormone; ACTH, adrenocorticotrophic hormone; CORT, corticosterone; CRFR1, corticotropin releasing factor receptor 1; 5-HT, 5-hydroxytryptamine; GDNF, glial cell line-derived neurotrophic factor; GFRα3, GDNF family receptor α3; LTP, long-term potentiation; IL-6, interleukin-6; IL-1β, interleukin-1β; TNF-α, tumor necrosis factor alpha; PPD, preprodynorphin; KOR, κ receptor; OFQ, orphanin-FQ; DRG, dorsal root ganglion; PKR1, prokineticin1 receptor; PK2, prokineticin2; PKR2, prokineticin2 receptor; RMP, resting membrane potential; ST25, *Tianshu;* ST36, *Zusanli*; ST37, *Shangjuxu*; NLRP6, NOD-like receptor family pyrin domain containing 6, IL-8, interleukin-8; IL-18, interleukin-18; TPSP, tryptase; TRPV1, transient receptor potential vanilloid 1; 5-HT3R, 5-hydroxytryptamine 3 receptor; 5-HT4R, 5-hydroxytryptamine 4 receptor; NGF, nerve growth factor; TrkA, tyrosine kinase receptor A; BDNF, brain-derived neurotrophic factor; TrkB, tyrosine kinase receptor B; SP, substance P; CGRP, calcitonin gene related peptide; VIP, vasoactive intestinal peptide; ITLN1, intestine lectin 1; MUC2, mucin-2; 5-HIAA, 5-hydroxyindoleacetic acid; SERT, serotonin transporter; RELMβ, resistance-like molecule β; TPH, tryptophan hydroxylase; M_3_R, M3 muscarinic acetylcholine receptor; SS, somatostatin.

The current fundamental research on the mechanisms underlying acupuncture and moxibustion analgesia primarily centers around examining cellular and molecular alterations within local tissue of the affected area, DRG neurons, and the central nervous system. This research explores the intricate interactions among inflammatory factors, neurotransmitters, and ion channels. Moreover, we specifically emphasize understanding multidimensional aspects of pain, including pain sensation, pain cognition, and pain emotion, which are also evident in the context of the IBS model. At the peripheral level, acupuncture may regulate pain signal production in IBS through several pathways: (1) By improving the inflammatory environment, blocking NGF/TrkA pathway, reducing the number of MCs and inhibiting their degranulation, TRPV1 ion channel, which modulates pain development in intestinal nerve cells can be inhibited, Ca^2+^ inflow and neuron excitation can be reduced, and the release of SP, CGRP, and other neurotransmitters can be inhibited. (2) EA can also inhibit 5-HT secretion by EC cells via the Epac1/Piezo2 pathway, and downregulate 5-HT3A receptor expression on intestinal sensory nerve cells, promoting the expression of 5-HT4 receptor with an anti-inflammatory effect, thereby preventing SP and CGRP release, and weaken pain signals. (3) Downregulated the expression of BDNF/TrkB, GDNF/GFRα3 pathway and NGF in intestinal EGCs promoted the stability of EGCs. (4) When the local signal is transmitted to the intestinal sensory nerve endings, the peripheral nerve sensitization is decreased, and acupuncture weakens the pain signal by reducing the excitability of gut-related DRG neurons, which may be related to the endogenous opioid pathway and the sympathetic nerve germination in the DRG. At the central level, besides exploring the well-established central opioid pathway, acupuncture research has also focused on examining its impact on reducing the expression of NMDAR (*N*-methyl-D-aspartate receptor) on the neuronal membrane within the dorsal horn of the spinal cord and the ACC brain region associated with the colon. This reduction in NMDAR expression helps prevent the transmission of pain signals, thereby attenuating the pain sensation. The role of central glial cells can also not be ignored. Acupuncture reduced the expression of P2X7 and PKs receptors in spinal cord astrocytes and GFAP in the optic cortex and ACC astrocytes, suggesting that neurons and glial cells in the spinal dorsal horn and ACC can mediate the effects of acupuncture in suppressing the formation of IBS pain perception. Furthermore, the brain-gut axis, a newly explored communication pathway between the central nervous system and the peripheral gut, has gained attention in recent years. A few studies have shifted their focus toward investigating whether acupuncture and moxibustion alleviate depression and anxiety by targeting the HPA axis, subsequently mitigating VH exacerbation. These studies indicate that acupuncture and moxibustion techniques can effectively reduce the expression of CRH, ACTH, and CORT within the HPA axis, at the same time simultaneously improving visceral stress. It is plausible that emotional regulation mediates this process (Figure 2).

Nevertheless, the present research has yet to establish a comprehensive and interconnected mechanism chain between the upstream and downstream components. For instance, most experiments detect the content of SP, CGRP, and other neuropeptides in the colon. Nevertheless, it remains unclear whether all the increased neuropeptides are solely secreted by intestinal sensory neurons and glial cells. Additionally, whether changes in local colon indicators are due to blocking the incoming pain signal at an early stage or the effect of analgesic signals originating from higher centers is unclear. Furthermore, investigating the specific interactions between various pathways can pave the way for future research. Additionally, IBS is considered a functional imbalance between the sympathetic and parasympathetic nervous systems. A previous study revealed that EA or transcutaneous electrical nerve stimulation therapy via somatic sympathetic reflex can restore this balance (Takahashi, 2011). However, studies on the effects of acupuncture on the sympathetic and parasympathetic nerves are inadequate, and only one paper mentioned that EA suppressed the germination of sympathetic nerve fibers toward the cell layer of DRG sensory neurons. Investigating the impact of acupuncture on the autonomic nervous system is a potential avenue for future research. Interestingly, one study on IBS rats introduced transcutaneous electrical nerve stimulation electrodes at non-gastrointestinal acupoints located on the left and right upper chest and upper back. Surprisingly, the study revealed that stress-induced VH could be alleviated via somatic sympathetic reflex, even in the absence of direct acupoint stimulation. Notably, the most significant reduction in VH was observed with stimulation applied to the left clavicular and left scapula regions compared to other experimental groups (Huang et al., 2019). This study provides a novel perspective that prompted investigation as to whether acupoint specificity and stimulation affect the analgesic effect of VH in IBS models. Additionally, there could be secondary changes in brain activity after pain hypersensitivity caused by nociceptive stimulation of IBS. Nevertheless, minimal attention has been devoted to investigating the impact of acupuncture on the brains of IBS rats despite three studies focusing on the ACC and two studies examining the medulla oblongata. Perhaps future acupuncture research, coupled with brain electrophysiology and imaging techniques to aid detection methods, can act as a novel avenue to investigate the analgesic mechanism and central neural loops of IBS.

Gut microbiota is responsible for the development of chronic pain in IBS. Studies indicate that gut microbiota communicates with the central nervous system via at least 3 parallel and interacting channels, involving the neural, endocrine and immune signaling mechanisms. The brain can influence the community structure and function of intestinal microbiota via the autonomic nervous system, and by modulating regional intestinal peristalsis, intestinal transport and secretion, and intestinal permeability, as well as possibly through the direct regulation of microbial gene expression to control hormones secretion (Martin et al., 2018). Acupuncture and moxibustion are currently known to improve the intestinal microbiota through the brain-gut axis. Studies on IBS have also shown that acupuncture and moxibustion can simultaneously alleviate VH, reduce AWR, and optimize the intestinal microbiota environment (by increasing the content of lactic acid bacteria). These changes in gut microbiota highly correlate with the proliferation and differentiation of intestinal immune cells (Zhou et al., 2020). The regulatory effect on intestinal flora may potentially be another strategy that acupuncture alleviates VH.

Summarily, the findings suggest that the evaluation criteria employed to gauge the impacts of acupuncture and moxibustion on VH in IBS rats predominantly center on distinct acupoints and intervention variables. However, the studies presented thus far have only lightly touched upon the molecular-level connections between the upstream and downstream elements. Consequently, there exists a requirement for further investigation aimed at uncovering the molecular mechanisms that underlie the observed effects of acupuncture and moxibustion. This pursuit will enhance our comprehension of the therapeutic capabilities of these modalities for addressing VH in IBS.

## Author contributions

ZX, YG, and XL: conceptualization and supervision. YY and JW: methodology, data collection, and article writing. CZ, YRW, JC, MD, and YZW: data collection, analysis, and preparation of the tables. ZX, XL, and MDZ: review and editing. All authors contributed to the article and approved the submitted version.
